# Differences in ICSI utilization rates among states with insurance mandates for ART coverage

**DOI:** 10.1186/s12958-021-00856-4

**Published:** 2021-11-30

**Authors:** Pavel Zagadailov, Kyung S. Cho, David B. Seifer

**Affiliations:** 1 Clinical Outcomes Research Group, CORG LLC, 178 Meadow Brook Rd, Grantham, NH 03753 USA; 2 Department of Statistics, Columbia University, 1255 Amsterdam Ave, New York, NY 10027 USA; 3 Department of Obstetrics and Gynecology, Yale University School of Medicine, 333 Cedar St, New Haven, CT 06510 USA

**Keywords:** Assisted reproductive technology, In-vitro fertilization, State insurance mandates, Intracytoplasmic sperm injection, Utilization rates, Live birth rate, Male factor

## Abstract

**Background:**

Assisted reproductive technology (ART) insurance mandates promote more selective utilization of ART clinic resources including intracytoplasmic sperm injection (ICSI). Our objective was to examine whether ICSI utilization differs by state insurance mandates for ART coverage and assess if such a difference is associated with male factor, preimplantation genetic testing (PGT), and/or live birth rates.

**Methods:**

In this retrospective analysis of the Centers for Disease Control (CDC) data from 2018, ART clinics in ART-mandated states (*n* = 8, AR, CT, HI, IL, MD, MA, NJ, RI) were compared individually to one another and with non-mandated states in aggregate (*n* = 42) for use of ICSI, male factor, PGT, and live birth rates. ANOVA was used to evaluate differences between ART-mandated states and non-mandated states. Individual ART-mandated states were compared using Welch t-tests. Statistical significance was determined by Bonferroni Correction.

**Results:**

There were significant differences in ICSI rates (%, mean ± SD) between MA (53.3 ± 21.3) and HI (90.7 ± 19.6), *p* = 0.028; IL (86.5 ± 18.7) and MA, *p* = 0.002; IL and MD (57.2 ± 30.8), *p* = 0.039; IL and NJ (62.0 ± 26.8), *p* = 0.007; between non-mandated states in aggregate (79.9 ± 19.9) and MA, *p* = 0.006, and NJ (62.0 ± 26.8), *p* = 0.02. Male factor rates of HI (65.8 ± 16.0) were significantly greater compared to CT (18.8 ± 8.7), IL (26.0 ± 11.9), MA (26.9 ± 6.6), MD (29.3 ± 9.9), NJ (30.6 ± 17.9), and non-mandated states in aggregate (29.7 ± 13.7), all *p* < 0.0001. No significant differences were reported for use of PGT and/or live birth rates across all age groups regardless of mandate status.

**Conclusions:**

ICSI use varied significantly among ART-mandated states while demonstrating no differences in live birth rates. These data suggest that the prevalence of male factor and the presence of a state insurance mandate are not the only factors influencing ICSI use. It is suggested that other non-clinical factors may impact the rate of ICSI utilization in a given state.

**Supplementary Information:**

The online version contains supplementary material available at 10.1186/s12958-021-00856-4.

## Background

Intracytoplasmic sperm injection (ICSI) is indicated for couples with a history of failed fertilization after conventional insemination or with severe male factor (MF) infertility. While ICSI is often used for the treatment of unexplained infertility and low oocyte yield, it has not been shown to improve clinical outcomes in couples with non-male infertility [1]. A recent randomized controlled trial demonstrated that ICSI did not improve the live birth rates (LBR) compared with conventional in-vitro fertilization (IVF) in couples without contributing MF [2]. Another study echoed the findings that ICSI does not improve the reproductive outcomes in an isolated group of advanced-age patients undergoing conventional insemination for non-male factor infertility [3]. Recent studies suggest that the use of ICSI should be limited to those with male factor infertility or a history of total fertilization failure due to uncertainties of potential adverse effects of ICSI [4]. Furthermore, conventional IVF and not ICSI should be used in non-male factor infertility in ART cycles with and without prenatal genetic testing (PGT) [5].

The original research examining the dramatic increase in ICSI use since 1995 demonstrated a greater use of ICSI for infertility that is not attributed to MF in mandated states [6]. This effort was followed by the report on ICSI use for non-male factor indications developed by the Practice Committee of the American Society for Reproductive Medicine (ASRM) and the Society for Assisted Reproductive Technology (SART) [7], with most recent update published in 2020 [1]. These efforts contributed to more stringent criteria for reimbursement of ICSI by insurance providers in ART-mandated states, potentially led to decrease of ICSI use for non-male-factor infertility cycles in ART-mandated states [8] and were associated with improved clinical pregnancy (CPR) and LBR, as well as greater elective single-embryo transfers (eSET) rates and lower twin rates [9].

Sixteen U.S. states have now passed laws that require insurers to offer coverage for infertility diagnosis and treatment. Before 2018, eight states (AR, CT, HI, IL, MA, MD, NJ, RI) had ART mandates. Four states (IL, MA, NJ, and RI) have included ICSI as a covered benefit [10–13].

Our previous studies have suggested that ICSI may still be overutilized and not accompanied by increase in MF or improved LBR [14, 15]. Additionally, in ART-mandated states, lower ICSI rates were accompanied by a positive correlation with LBR. Such findings suggested that ART mandates may promote more selective utilization of ART clinic resources [9]. However, these previous analyses were comparing ART-mandated versus non-mandated states in aggregate.

The present study was designed to evaluate the differences in utilization of ICSI between individual ART-mandated states and to compare these rates with non-mandated states in aggregate. Furthermore, to better understand some of the underlying factors contributing to differences in ICSI use among individual ART-mandated states, we examined the frequency of male factor, PGT and singleton live birth rates among these states.

## Methods

### Data source

This retrospective analysis was conducted using the National Assisted Reproductive Technology Surveillance System (NASS), maintained by the Centers for Disease Control and Prevention (CDC). A publicly available NASS dataset for 2018 was downloaded from the CDC website [16]. Clinics within this yearly report were grouped by ART-mandated state, then evaluated individually and compared to a group of non-mandated states in aggregate.

### Study design and outcomes

We evaluated eight ART-mandated states (AR, CT, HI, IL, MD, MA, NJ, RI) individually, comparing them to one another as well as comparing each of them to the remaining non-mandated states (*n* = 42, 382 ART clinics) in aggregate. We hypothesized that the use of ICSI, frequency of male factor, PGT rates, and singleton live birth rates across all age groups significantly varied among each of the ART-mandated states. Age groups were categorized by the Society for Assisted Reproductive Technology (SART) groupings as < 35 years old, 35–37, 38–40, 41–42, and > 42 years old.

Only autologous, non-donor embryo transfers were included in this analysis. Frozen autologous and donor embryo transfers were excluded. Specific for each age group, ICSI utilization rates, frequency of male factor, PGT rates, and singleton live birth rates for individual ART-mandated states and non-mandated states combined in aggregate were evaluated. ICSI utilization rates and MF infertility rates are examined to assess the potential overutilization of ICSI in ART-mandated states if any.

### Statistical analysis

Statistical analysis was performed utilizing R (version 3.5.1, R Core Team, University of Auckland, New Zealand). Analysis of variance (ANOVA) was used to evaluate whether there were statistical differences among individual ART-mandated states and non-mandated states in aggregate. Individual states were compared in pairs using Welch t-tests to determine whether utilization of ICSI, male factor, PGT rates, and live birth rates per transfer were statistically different among ART-mandated states and non-mandated states in aggregate. Statistical significance was determined after multiple testing adjustments using Bonferroni Correction.

## Results

Results are organized by age group.

*< 35 years old* There were significant differences between ICSI utilization rates (%, mean ± SD) of MA (53.3 ± 21.3) and HI (90.7 ± 19.6), *p* = 0.028, 95% CI [11.4, 63.4]; IL (86.5 ± 18.7) and MA, *p* = 0.002, 95% CI [14.5, 51.9]; IL and MD (57.2 ± 30.8), *p* = 0.04, 95% CI [2.9, 61.5]; IL and NJ (62.0 ± 26.8), *p* = 0.007, 95% CI [8, 41.1]; non-mandated states combined (79.9 ± 19.9) and MA, *p* = 0.006, 95% CI [8.8, 44.4]; non-mandated states and NJ (62.0 ± 26.8), *p* = 0.02, 95% CI [3, 32.9]. Male factor rates of HI (65.8 ± 16.0) were greater compared to CT (18.8 ± 8.7), IL (26.0 ± 11.9), MA (26.9 ± 6.6), MD (29.3 ± 9.9), NJ (30.6 ± 17.9) and non-mandated states (29.7 ± 13.7), all *p* < 0.0001, 95% Cl’s are [29.7, 64.3], [23, 56.6], [22.1, 55.8], [19.2, 53.9], [17.9, 52.7], [19.3, 52.9] respectively (Table 1).

**Table 1:** Age group < 35

States	Live Birth Rate (%, mean ± SD)	ICSI Rate (%, mean ± SD)	PGT Rate (%, mean ± SD)	Male Factor Rate (%, mean ± SD)
AR	30.7	70.2	0.8	18
CT	46.2 ± 11.7	72.1 ± 16.8	28.4 ± 17.1	18.8 ± 8.7
HI	45.1 ± 19.8	90.7 ± 19.6	25.8 ± 13.7	65.8 ± 16
IL	34.8 ± 12.8	86.5 ± 18.7	20.1 ± 21	26 ± 11.9
MD	39.9 ± 8.5	57.3 ± 30.8	11.3 ± 11.3	29.3 ± 9.9
MA	36.7 ± 6.7	53.3 ± 21.3	20 ± 24.2	26.9 ± 6.6
NJ	40.6 ± 9.1	62 ± 26.8	32.6 ± 22.4	30.6 ± 17.9
RI	33.1	55.1	7.2	23
non-mandated states	40.9 ± 10.6	79.9 ± 19.9	35.6 ± 28.6	29.7 ± 13.7

*35–37 years old* There were significant differences between ICSI utilization rates of MA (51.85 ± 19) and HI (87.7 ± 20), *p* = 0.0459, 95% CI [10.2, 61.5]; IL (84.7 ± 21) and MA, *p* = 0.0024, 95% CI [15.5, 50.2]; NJ (61.9 ± 24.6) and IL, *p* = 0.0327, 95% CI [5.8, 39.7]; non-mandated states combined (77 ± 20.2) and MA, *p* = 0.0135, 95% CI [9.2, 41.1] (Additional file 1).

*38–40 years old* There were significant differences between ICSI utilization rates of MA (54.5 ± 20.4) and IL (86 ± 11.6), *p* = 0.004, 95% CI [11.9, 48.6] (Additional file 2).

*41–42 years old and 42 > years old* There were no statistically significant differences in ICSI utilization rates in two age groups between ART-mandated and non-mandated states (Additional files 3, 4).

No statistically significant differences were reported for PGT rates and singleton live birth rates between ART-mandated and non-mandated states for all age groups.

## Discussion

These data demonstrate significant differences in ICSI utilization between individual ART-mandated states as well as compared to non-mandated state aggregate data. Covered infertility benefits in ART-mandated states vary dramatically (Table 2). Multiple factors may influence the decision of whether to use ICSI or conventional IVF. Clinic-specific policies, the discretion of treating physicians, and/or embryology laboratory personnel preference to use ICSI in patients with low oocyte yields, perceived poor oocyte quality or unexplained infertility are factors that can influence the use of ICSI. Additionally, insurance plans (i.e. Aetna, United, BCBS / WIN Fertility, etc.) can decline coverage of ICSI if the indication for ART is not a male factor. There could certainly be variability of such insurance policies/requirements between ART-mandated states.

**Table 2:** Comparison of insurance coverage for infertility diagnosis and treatment in ART-mandated states

ART-Mandated State (n/clinic)	Enacted (and Revised)	Diagnoses	Cycle type	Lifetime maximum	Costs of diagnostic tests	Medications	ICSI	Cryopreservation	Coverage of other ART procedures
Arkansas (*n* = 1)	1987 2011	2-years of infertility OR Endometriosis Tubal factor Male factor	Autologous only	$15.000	Not covered	Not covered	Not specified	Covered	Plan-dependent
Connecticut (*n* = 6)	1989 2005 2017	1-year of infertility	Not specified	3 IUI 2 IVF cycles	Covered	Covered, plan-dependent	Not specified	Not covered	Not specified
Hawaii (*n* = 6)	1989 2003	5-year of infertility OR Endometriosis Tubal factor Male factor	Autologous only	1 IVF cycle	Partially covered	Not covered	Not specified, plan-dependent	Covered, plan-dependent	Plan-dependent
Illinois (*n* = 26)	1991 1997	1-year of infertility	Autologous and Donor	6 IVF cycles	Covered	Covered	Covered	Covered, plan-dependent	Plan-dependent
Maryland (*n* = 7)	1985 2000	2-year of infertility OR Endometriosis Tubal factor Male factor	Autologous only	$100.000 3 IVF cycles	Covered	Not covered	Not specified, plan-dependent	Not covered	Not specified
Massachusetts (*n* = 8)	1987 2010	1-year of infertility for < 35 years old 6-months infertility ≥35 years old	Not specified	None	Covered, plan-dependent	Covered, plan-dependent	Covered	Covered	Plan-dependent
New Jersey (*n* = 19)	2001 2017	1-year of infertility for < 35 years old 6-months infertility ≥35–46 years old OR Tubal factor Male factor	Autologous and Donor	4 IVF cycles	Covered	Covered	Covered	Not covered	Not specified
Rhode Island (*n* = 1)	1989 2006 2017	1-year of infertility between 25 and 42 years old	Autologous only	$100.000	Covered	Covered	Covered	Covered, plan-dependent	Not specified

The two ART-mandated states with the highest utilization of ICSI were HI and IL. Remarkably, a large portion of the ART clinics in Hawaii (80%) and in Illinois (48%) reported > 75% ICSI usage, in contrast to no ART clinics in MA. In addition, HI reported dramatically higher male factor infertility rates (65.8%) than any other ART-mandated and non-mandated state. Furthermore, CT and MA showed a similar trend of lower ICSI use associated with more favorable clinical outcomes than reported in HI and IL. However, significantly greater male factor rates in HI than in other ART-mandated states and non-mandated states in aggregate may justify greater utilization of ICSI. This observation raises speculation of whether increased concern for potential poor, failed fertilization, unexplained infertility, or the perception of increased competition in specific geographic locations may have contributed to greater utilization of ICSI.

Interestingly, further analysis of the states with mandated ART coverage identified two states (IL and MA) with similar ART mandate structure and demographics but dramatically different ICSI utilization profiles. Both state mandates have a similar timeline for infertility diagnosis, covered cost of diagnostic tests, laboratory procedures that include ICSI, plan-dependent cost of medication and embryo cryopreservation (Table 2). Both states have heterogeneous, racially diverse populations within major metropolitan centers (Chicago and Boston) that are the homes of multiple academic teaching medical centers as well as many IVF private practices. However, IL had remarkably higher ICSI rates compared with ICSI rates of MA. Yet, in contrast, male factor diagnosis rates did not differ significantly between these two states, nor did PGT or singleton live birth rates. This broad difference in ICSI rates between IL and MA may partly be due to fewer clinics and thus a greater annual ART cycle volume per IVF clinic in MA than in IL. It is speculated that lower and more selective use of ICSI utilization in MA could contribute to greater uniformity and less variability in employing ICSI by MA clinics compared to those in IL.

Furthermore, several unique factors may explain why HI demonstrated the highest rate of ICSI among the ART-mandated states. Such factors contributing to increased ICSI utilization may include the limited number of ART clinics with significantly lower annual clinical volume, island-specific demographic distribution and a limited number of laboratory directors. Thus, we believe trends of ICSI use in HI may not be as representative nor as generalizable to ICSI and outcome rates of states in the continental U.S.

ICSI use varied significantly among the ART-mandated states while demonstrating no differences in live birth rates. This analysis of individual ART-mandated states suggests that the prevalence of male factor and the presence of a state insurance mandate are not the only factors influencing ICSI use. It is suggested that other possible non-clinical factors, such as the number of ART clinics in a given geographic area, clinic-specific policies, and/or patient/physician preferences, may impact the rate of ICSI utilization in a given state and will require further examination.

There are several strengths and limitations to this study. The primary strength is the use of the CDC dataset that incorporates > 98% of ART cycles performed in the U. S. The improvement in the reporting of the 2018 data set compared to previous years included outcomes specific for each age group. Live birth rates were reported per transfer and specific for fresh non-donor embryos resulting from ICSI use.

Limitations include the fact that states with single clinics (AR, RI) were excluded as the variance calculation was possible only for states with two or more clinics. Hence, we could not assess the impact of every state’s ART mandate. Provided by the CDC, male factor rates are “per clinic” and are not age group-specific nor do they include details regarding the specific types of male factor diagnosis. An additional limitation of this study is that semen parameters were not collected nor available from the CDC dataset to help better understand the origin of the greater rates of male factor in HI.

## Conclusions

ICSI use varied significantly among ART-mandated states while demonstrating no differences in live birth rates. These data suggest that the prevalence of male factor and the presence of a state insurance mandate are not the only factors influencing ICSI use. It is suggested that other non-clinical factors may impact the rate of ICSI utilization in a given state.

## Supplementary Information

**Additional file 1: Appendix A**: Age group 35–37

**Additional file 2: Appendix B**: Age group 38–40

**Additional file 3: Appendix C**: Age group 41–42

**Additional file 4: Appendix D**: Age group ≥42

## Data Availability

NASS datasets for 2018 are publicly available from the CDC website: https://www.cdc.gov/art/nass/ See reference [10].

## Abbreviations

ANOVA: Analysis of variance
ART: Assisted reproductive technology
CDC: Centers for disease control and prevention
ICSI: Intracytoplasmic sperm injection
LBR: Live birth rate
MF: Male factor
NASS: National assisted reproductive technology surveillance system
PGT: Preimplantation genetic testing
SART: Society for assisted reproductive technology
