# Supplementary material for: Differences in ICSI utilization rates among states with insurance mandates for ART coverage
Source: Reprod Biol Endocrinol. 2021 Nov 30;19:174. doi: 10.1186/s12958-021-00856-4 (PMC8630859; doi:10.1186/s12958-021-00856-4)
Supplement: Supplementary file 1 — Additional file 1: Appendix A: Age group 35–37 [file 12958_2021_856_MOESM1_ESM.docx]

Appendix A: Age group 35 – 37

| **States** | **Live Birth Rate** (%, mean ± SD) | **ICSI Rate**  (%, mean ± SD) | **PGT Rate**  (%, mean ± SD) |
| --- | --- | --- | --- |
| AR | 25.8 | 71.6 | 5.4 |
| CT | 46.4 $\pm$ 9.6 | 72.2 $\pm$ 23.3 | 37.6 $\pm$ 25.1 |
| HI | 35.8 $\pm$ 9.7 | 87.7 $\pm$ 20 | 28.6 $\pm$ 20.3 |
| IL | 34.5 $\pm$ 14.2 | 84.7 $\pm$ 21 | 23.9 $\pm$ 21.9 |
| MD | 34.6 $\pm$ 4.8 | 74.2 $\pm$ 13.6 | 18.7 $\pm$ 15.6 |
| MA | 35.5 $\pm$ 4.4 | 51.9 $\pm$ 19 | 23.7 $\pm$ 25.2 |
| NJ | 35.1 $\pm$ 12.1 | 62 $\pm$ 24.6 | 38.4 $\pm$ 21.8 |
| RI | 26.7 | 49.7 | 9.1 |
| non-mandated states | 37.8 $\pm$ 12.1 | 77 $\pm$ 20.2 | 41.8 $\pm$ 28.82 |
