# Supplementary material for: Differences in ICSI utilization rates among states with insurance mandates for ART coverage
Source: Reprod Biol Endocrinol. 2021 Nov 30;19:174. doi: 10.1186/s12958-021-00856-4 (PMC8630859; doi:10.1186/s12958-021-00856-4)
Supplement: Supplementary file 2 — Additional file 2: Appendix B: Age group 38–40 [file 12958_2021_856_MOESM2_ESM.docx]

Appendix B: Age group 38 – 40

| **States** | **Live Birth Rate** (%, mean ± SD) | **ICSI Rate**  (%, mean ± SD) | **PGT Rate**  (%, mean ± SD) |
| --- | --- | --- | --- |
| AR | 17.4 | 75.9 | 10.3 |
| CT | 39.5 $\pm$ 17 | 70.3 $\pm$ 20.3 | 44.23 $\pm$ 21.6 |
| HI | 22.8 $\pm$ 14.3 | 84.2 $\pm$ 18.2 | 29.4 $\pm$ 13.2 |
| IL | 30 $\pm$ 10.6 | 86 $\pm$ 11.6 | 29.27 $\pm$ 22 |
| MD | 25.8 $\pm$ 6.4 | 70.5 $\pm$ 20.9 | 25.06 $\pm$ 22.9 |
| MA | 24.3 $\pm$ 6.7 | 54.5 $\pm$ 20.4 | 28.55 $\pm$ 25.1 |
| NJ | 31.4 $\pm$ 13.4 | 65 $\pm$ 20.3 | 36.62 $\pm$ 24.2 |
| RI | 27.8 | 56.2 | 8.6 |
| non-mandated states | 33.9 $\pm$ 12.9 | 75.6 $\pm$ 20 | 44.3 $\pm$ 27.7 |
