# Supplementary material for: Differences in ICSI utilization rates among states with insurance mandates for ART coverage
Source: Reprod Biol Endocrinol. 2021 Nov 30;19:174. doi: 10.1186/s12958-021-00856-4 (PMC8630859; doi:10.1186/s12958-021-00856-4)
Supplement: Supplementary file 3 — Additional file 3: Appendix C: Age group 41–42 [file 12958_2021_856_MOESM3_ESM.docx]

Appendix C: Age group 41 – 42

| **States** | **Live Birth Rate** (%, mean ± SD) | **ICSI Rate**  (%, mean ± SD) | **PGT Rate**  (%, mean ± SD) |
| --- | --- | --- | --- |
| AR | NA | NA | NA |
| CT | 25.1 $\pm$ 4.1 | 73.3 $\pm$ 17.6 | 44 $\pm$ 32 |
| HI | NA | 79.3 $\pm$ 26.1 | 20.2 $\pm$ 2.7 |
| IL | 21.6 $\pm$ 11.2 | 84.4 $\pm$ 11.2 | 27.4 $\pm$ 18 |
| MD | 15.9 $\pm$ 9.5 | 72 $\pm$ 19 | 22.2 $\pm$ 16.2 |
| MA | 19.5 $\pm$ 4.3 | 53 $\pm$ 23.1 | 15.7 $\pm$ 7.8 |
| NJ | 25.8 $\pm$ 19.4 | 65.4 $\pm$ 21 | 39.7 $\pm$ 26.1 |
| RI | 16.3 | 60.7 | 14.3 |
| non-mandated states | 26.1 $\pm$ 13.8 | 68.7 $\pm$ 22.3 | 42.8 $\pm$ 26.6 |
