# Supplementary material for: Differences in ICSI utilization rates among states with insurance mandates for ART coverage
Source: Reprod Biol Endocrinol. 2021 Nov 30;19:174. doi: 10.1186/s12958-021-00856-4 (PMC8630859; doi:10.1186/s12958-021-00856-4)
Supplement: Supplementary file 4 — Additional file 4: Appendix D: Age group ≥42 [file 12958_2021_856_MOESM4_ESM.docx]

Appendix D: Age group ≥ 42

| **States** | **Live Birth Rate** (%, mean ± SD) | **ICSI Rate**  (%, mean ± SD) | **PGT Rate**  (%, mean ± SD) |
| --- | --- | --- | --- |
| AR | NA | NA | NA |
| CT | 17.4 | 65.2 $\pm$ 24 | 38.9 $\pm$ 24.5 |
| HI | NA | 77.1 $\pm$ 13.8 | 42.7 $\pm$ 6.6 |
| IL | 14.2 $\pm$ 2.4 | 79.9 $\pm$ 12 | 25.8 $\pm$ 16.7 |
| MD | 9.2 | 73.7 $\pm$ 6.9 | 13.2 $\pm$ 7.1 |
| MA | 7.1 $\pm$ 1.8 | 54.1 $\pm$ 13.6 | 13.3 $\pm$ 11 |
| NJ | 15.5 $\pm$ 17.6 | 56.5 $\pm$ 15.6 | 28.6 $\pm$ 22.2 |
| RI | NA | 70 | 3.3 |
| non-mandated states | 10.4 $\pm$ 9.6 | 63.3 $\pm$ 22.7 | 34.9 $\pm$ 27.2 |
